# Spatial Distribution of Gestational Syphilis in Brazil: Socioeconomic and Health Services Inequalities

**DOI:** 10.4269/ajtmh.22-0449

**Published:** 2023-05-15

**Authors:** Janmilli da Costa Dantas, Cristiane da Silva Ramos Marinho, Yago Tavares Pinheiro, Maria Ângela Fernandes Ferreira, Richardson Augusto Rosendo da Silva

**Affiliations:** ^1^ Graduate Program in Collective Health, Federal University of Rio Grande do Norte, Natal, Brazil;; ^2^ Graduate Program in Collective Health, Faculty of Health Sciences of Trairi, Federal University of Rio Grande do Norte, Santa Cruz, Brazil;; ^3^ Graduate Program in Collective Health, Department of Dentistry, Federal University of Rio Grande do Norte, Natal, Brazil;; ^4^ Graduate Program in Collective Health, Health Sciences Center, Federal University of Rio Grande do Norte, Natal, Brazil

## Abstract

We aimed to analyze the spatial distribution of gestational syphilis from 2008 to 2018 in Brazil and identify correlations with socioeconomic and health-care aspects. This ecological study used municipalities of Brazil as the unit of analysis. Data collection took place between June and July 2021. Data were extracted for 2008 to 2018, and information on the epidemic in animals in the country was obtained from data records. The gestational syphilis detection rate was the dependent variable, and the independent variables were the Municipal Human Development Index, the proportion of doctors per inhabitant in primary health care (PHC), and the percentage of PHC coverage. The data went through an aggregation process in 482 immediate regions of urban articulation. The global Moran’s I index and the local spatial correlation indicator detected territorial clusters using GeoDa software. The gestational syphilis detection rate was distributed unevenly in the immediate regions of urban articulation between 2008 and 2018, and presented a negative spatial correlation with the Municipal Human Development Index (Moran’s I = −0.243, *P* ≤ 0.05), the percentage of PHC coverage (Moran’s I = −0.163, *P* ≤ 0.05), and the proportion of doctors per inhabitants in PHC (Moran’s I = −0.164, *P* ≤ 0.05). Socioeconomic inequalities, mainly related to the availability of human resources and access to health services, are correlated with the spatial distribution of gestational syphilis in Brazil. Investments in social policies and strengthening of PHC are essential for controlling gestational syphilis.

## INTRODUCTION

The number of cases of syphilis increased in recent years and has become a global public health problem even with diagnosis and treatment protocols. Syphilis is transmitted sexually and vertically, affects the health and lives of many people worldwide, and impacts reproductive and child health directly. It may cause abortion, stillbirth, premature birth, neonatal death, and early or late congenital manifestations.^1^

Studies estimate that more than 11 million new cases of syphilis occur worldwide annually, with high incidence rates in Latin America, Africa, and Asia.^2^ In Brazil, syphilis reemerged and was declared an epidemic in 2016.^3^ In 2020, 61,441 cases of gestational syphilis (GS) (detection rate, 21.6/1,000 live births), 22,065 cases of congenital syphilis (incidence rate, 7.7/1,000 live births), and 186 deaths were reported to the Notifiable Diseases Information System (SINAN).^4^

In 2014, the Pan American Health Organization created a committee to validate the vertical transmission of syphilis and HIV. For this, the committee recommends that 95% of pregnant women have access to at least one prenatal consultation and, if necessary, be tested and treated for syphilis. The committee also certificates countries that reach this goal; 11 have already been certified.^5^ Disease control requires knowledge regarding its territorial distribution and dispersion to evaluate, plan, and support clinical decision making, health management, and policies.^6^ Although some studies have analyzed the correlations between GS, socioeconomic factors, and health services in Brazil, the analysis of spatial distribution and factors correlated with GS throughout the country are scarce and restricted to states or cities.

In this context, the techniques developed in spatial analysis are tools for identifying areas of greater epidemiological pressure and associating the studied phenomenon with social and economic factors as well.^7^ When studying health care, it is important to consider the location and characteristics of where people seek their care. This is because the place where an individual lives or works should be considered a potential determinant of the disease. Thus, epidemiological research has been developed that involves geocoding, distance estimation, residential mobility, linking of records and data integration, spatial grouping and space–time, small-area estimation, and Bayesian applications for disease mapping. Linked to spatial analysis, geographic information systems have applicability in disease mapping, rate smoothing, cluster or hot spot analysis, and spatial modeling.^8^

The analysis of GS focusing on geographic location and establishing relationships with external factors may also reveal poorly explored results that may help control the disease in specific locations. Our study is relevant mainly because of spatial analysis, which identifies the spatial distribution of GS and reveals high-risk clusters of the disease using parameters related to socioeconomic conditions, human resources, and access to health services. Countries with contexts similar to Brazil may also benefit from our study because the results may improve health services and help reduce GS.

Our study aimed to analyze the spatial distribution of GS between 2008 and 2018 in Brazil, and to identify correlations with socioeconomic and health-care aspects.

## MATERIALS AND METHODS

For this ecological study, which uses secondary data from public domains, we selected immediate regions of urban articulation (IRUAs),^9^ proposed by the Brazilian Institute of Geography and Statistics (IBGE), as the unit of analysis.

Brazil has 5,565 cities and 27 states (including the Federal District), according to the 2010 census. The country is divided into five macro-regions: North, Northeast, Midwest, South, and Southeast. The IBGE adopts models of regional division, making the urban structure a fundamental element of space organization. This regionalization process is constructed from the definition of criteria that distinguish the regions of urban articulation (RUAs), using as references the Brazilian urban network, the hierarchy of its centers, the influence of urban areas, information from public and private administrations, and consumption of goods and services. They make the urban network compatible with zonal features, contiguous and without overlapping, commanded by a city that polarizes an area of influence of its own. It identifies regions where cities articulate in three scales of RUAs: expanded regions, intermediate regions and immediate regions.^9^

The IRUAs largely reflect the area lived by the population and its daily commute to supply and search for everyday goods and services. In this urban–regional division model, composed of 482 territories, the region is contiguous and each municipality belongs to a single territorial unit, with boundaries that are not restricted to state borders. Each region has a core municipality that exerts influence in macro-regional terms over the other municipalities that comprise it through the supply of highly complex goods and services.

Our sample comprised reported cases of GS by city of residence from January 1, 2008 to December 31, 2018. Data were obtained from the following public databases: the Department of Informatics of the Unified Health System, SINAN, Live Birth Information System, National Registry of Health Establishments, the United Nations Development Program, IBGE, and Primary Care e-Manager. The Primary Care e-Manager is the database that gives access to several information systems of primary health care (PHC) in Brazil. It was used to extract data on PHC coverage in Brazil. The IBGE estimated that 208,494,900 inhabitants occupied an area of 8,510,820,623 km^2^ in 2018.^10^

Data were collected from June to July 2021. The GS detection rate was the dependent variable, and the independent variables were the Municipal Human Development Index (MHDI), the proportion of doctors per inhabitant in PHC, and the percentage of PHC coverage. Data from outcome variables, human resources, infrastructures, and PHC coverage were collected from 2008 to 2018, and data related to social and economic conditions were collected exclusively from 2010. Indicators were created by weighting data to minimize possible discrepancies resulting from different population sizes (Supplemental Table 1).

An exploratory analysis of spatial data was performed using thematic maps in the GeoDa 1.14 (University of Chicago, EUA) program to observe the distribution of variables. Subsequently, the global Moran’s I index and the local spatial association indicator (LISA)^11^ were applied. The global Moran’s I coefficient verified the spatial autocorrelation between variables, and results ranged from −1 (inverse correlation) to +1 (direct correlation); a value of zero represented no correlation.

The global spatial autocorrelation and local correlation coefficients were considered significant at *P* ≤ 0.05.^12^ The LISA values for the 482 IRUAs were submitted to the Moran scatterplot, adopting a statistical significance of 95% (*P* ≤ 0.05). Regions of urban articulation were divided into five classes: high–high (the unit and its neighbors had values greater than the average of the set), high–low (high value for the unit and low average values for its neighbors), low–high (the unit had a low value for a variable, whereas its neighbors had values greater than the average of the set), low–low (values of the unit and its neighbors were below the average of the set), and not significant (the unit has no defined relationships with its neighbors).^12^^,^^13^

The LISA bivariate analysis was performed using GeoDa 1.14 software to assess the spatial correlation between the dependent variable (GS detection rate) and each independent variable (MHDI, the proportion of physicians per inhabitant in PHC, and the percentage of PHC coverage). Through this analysis, the Moran local index, maps, and correlation scatterplot were generated. Moran I statistical significance was verified by a test with 99 random permutations. In this bivariate spatial correlation, five types of spatial clusters are observed: nonsignificant (territories that did not form clusters because the differences were not significant), high–high (areas formed by IRUAs with high frequencies of the dependent variable and high frequencies of the independent variables), low–low (areas formed by IRUAs with low frequencies of the dependent variable and low frequencies of the independent variables), high–low (areas formed by IRUAs with high frequencies of the dependent variable and low frequencies of the independent variables), and low–high (areas formed by IRUAs with low frequencies of the dependent variable and high frequencies of the independent variables).^11^

## RESULTS

**Figure 1.**
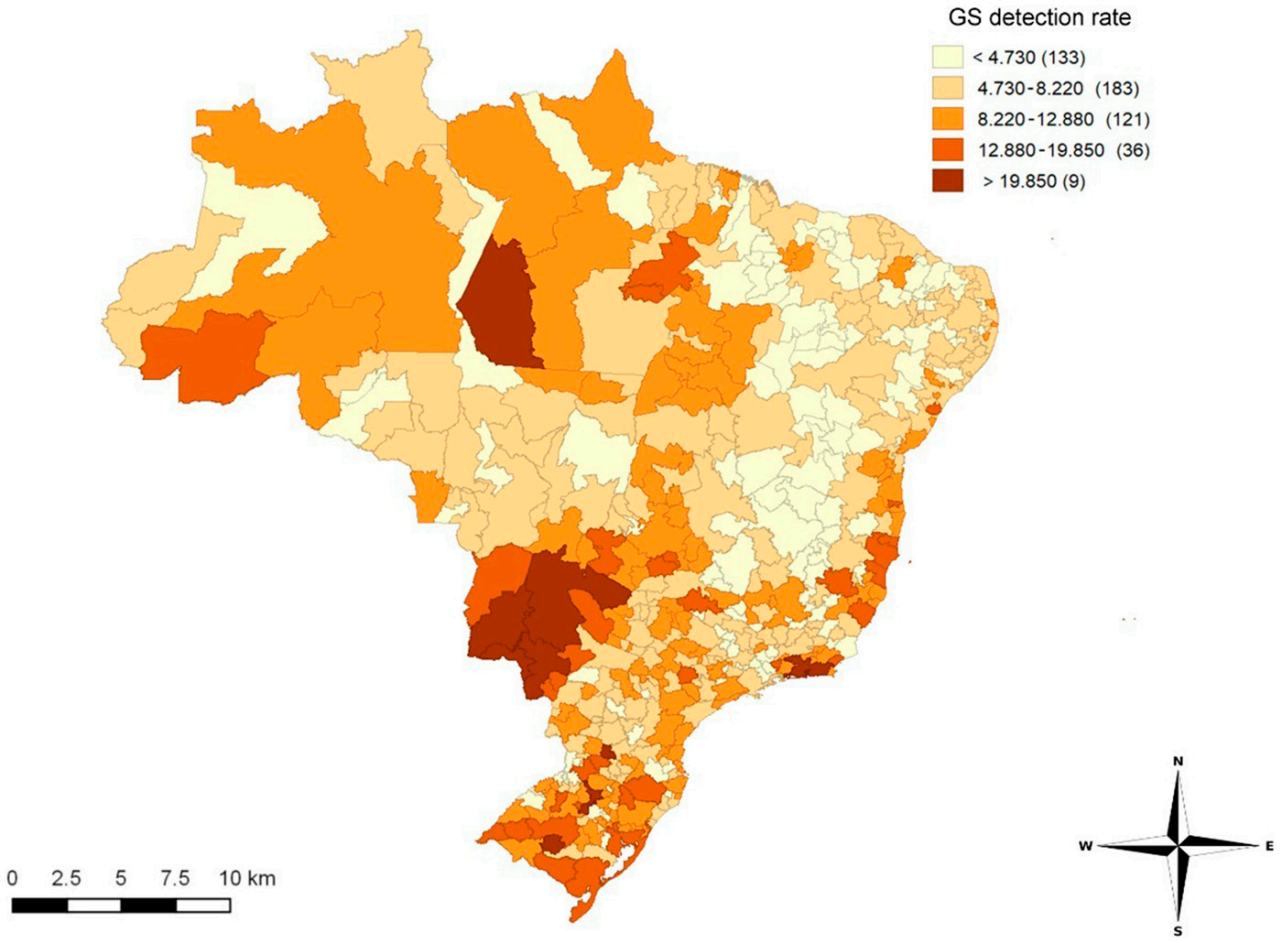
Spatial distribution of gestational syphilis (GS) detection rate (exploratory map) according to immediate regions of urban articulation. Values in parentheses represent the number of immediate regions of urban articulation with a *P* value ≤ 0.05. Brazil, 2008 to 2018.

The SINAN recorded 296,523 cases of GS in Brazil between 2008 and 2018. The largest number of notifications was observed in 2018 (59,006 cases). The GS detection rate increased (2.49/1,000 live births in 2008 to 20.04/1,000 live births in 2018) and was distributed unevenly in IRUAs from 2008 to 2018 (Moran’s I = 0.387; *P* ≤ 0.01) (Figure 1).

**Figure 2.**
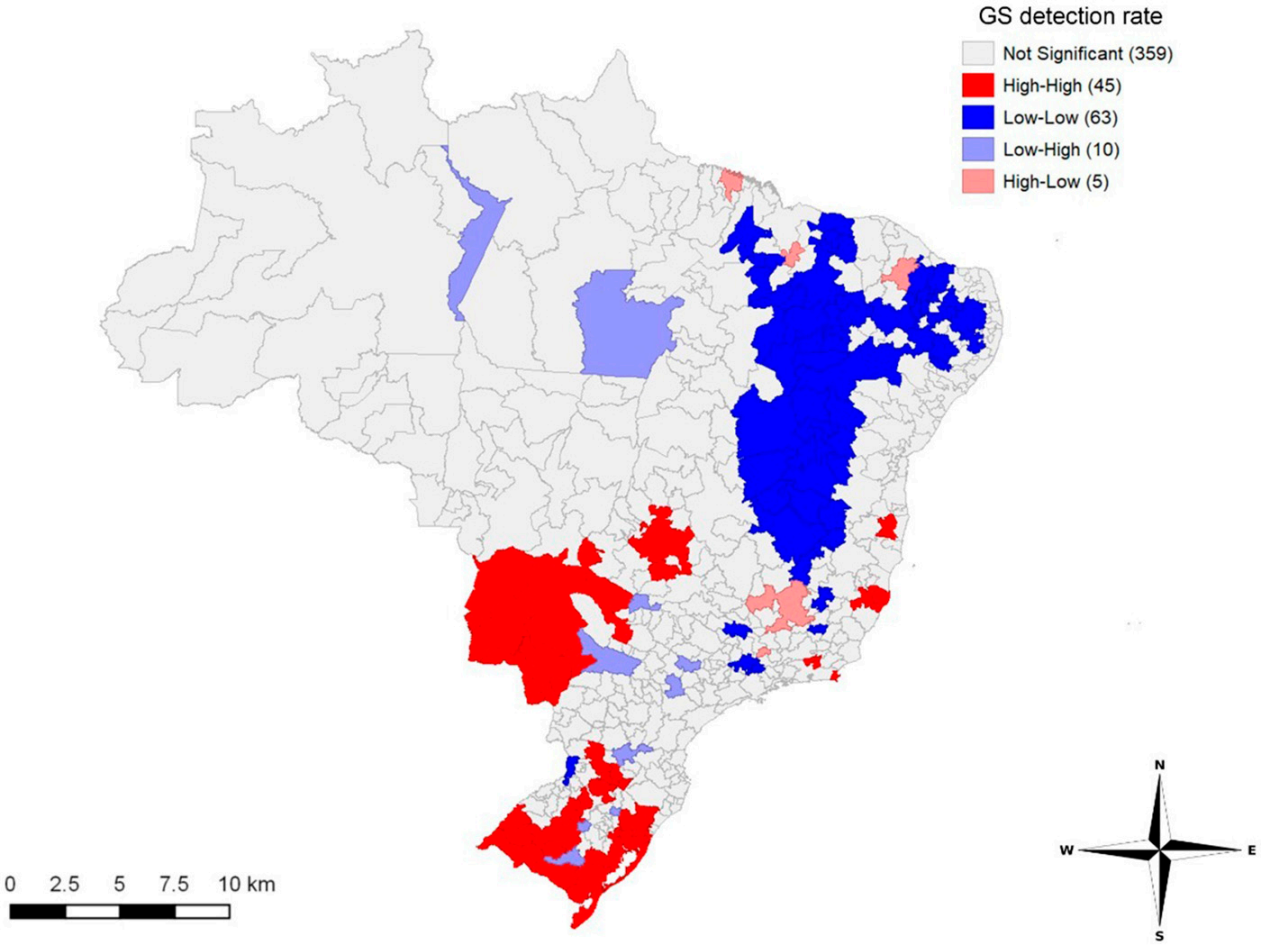
Spatial distribution of gestational syphilis (GS) detection rate according to immediate regions of urban articulation. Values in parentheses represent the number of immediate regions of urban articulation with a *P* value ≤ 0.05. Brazil, 2008 to 2018.

Figure 2 highlights high–high clusters in the South and part of the Midwest. The central part of the Northeast presented low–low clusters (Moran’s I = 0.396, *P* ≤ 0.05).

**Figure 3.**
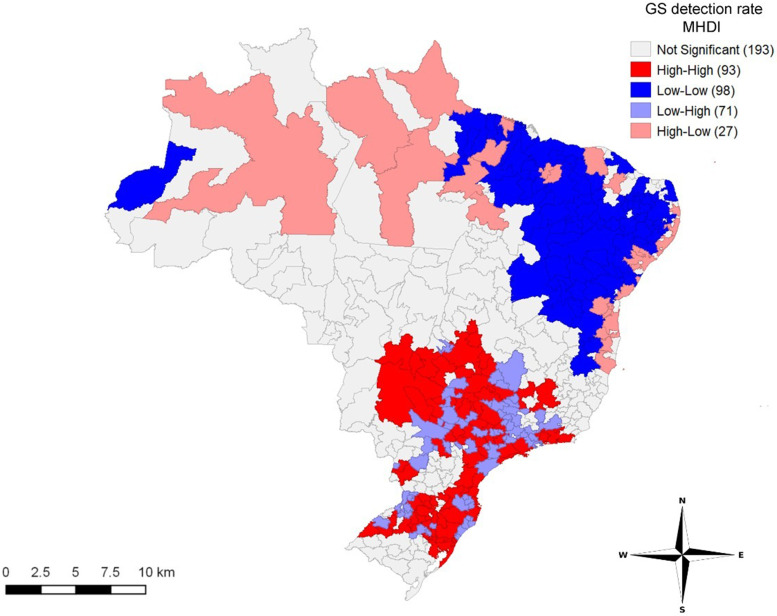
Spatial distribution of gestational syphilis (GS) detection rate and Municipal Human Development Index (MHDI) in the immediate regions of urban articulation. Values in parentheses represent the number of immediate regions of urban articulation with a *P* value ≤ 0.05. Brazil, 2008 to 2018.

Figure 3 shows the spatial distribution of the GS detection rate and the MHDI for the 482 IRUAs from 2008 to 2018. According to the LISA method, GS correlated inversely with MHDI (Moran’s I = −0.243, *P* ≤ 0.05). The presence of clusters in the North highlights the low human development and high number of cases of GS. Although the South, Southeast, and Midwest presented a high human development, cases of GS remained high.

**Figure 4.**
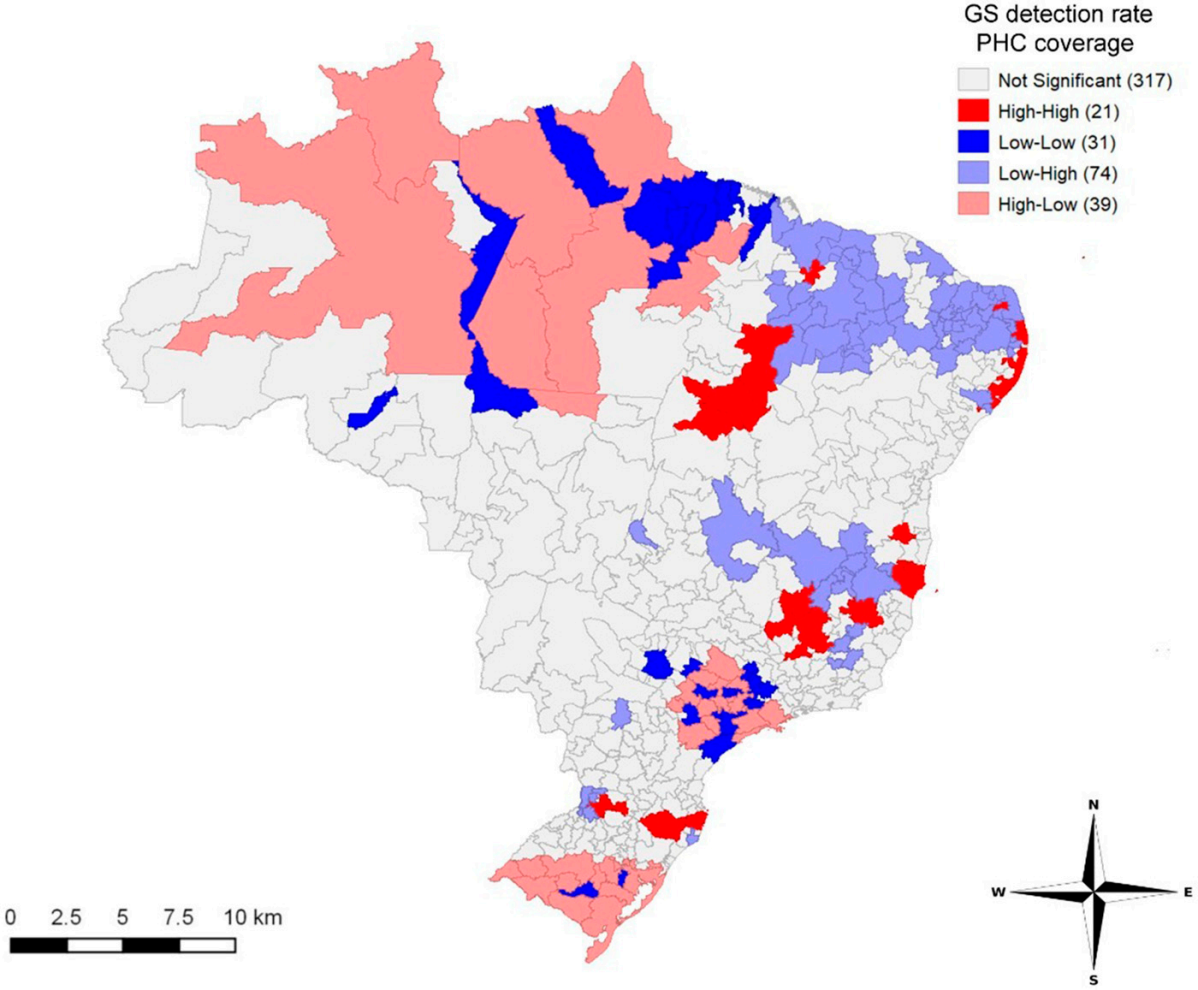
Spatial distribution of gestational syphilis (GS) detection rate and the percentage of primary health-care (PHC) coverage in immediate regions of urban articulation. Values in parentheses represent the number of immediate regions of urban articulation with a *P* value ≤ 0.05. Brazil, 2008 to 2018.

Correlations between the spatial pattern of the GS detection rate and the percentage of PHC coverage in IRUAs (Moran’s I = −0.163, *P* ≤ 0.05) are presented in Figure 4. Clusters with a high GS detection rate and low PHC coverage were observed in the North, and in the states of São Paulo and Rio Grande do Sul.

**Figure 5.**
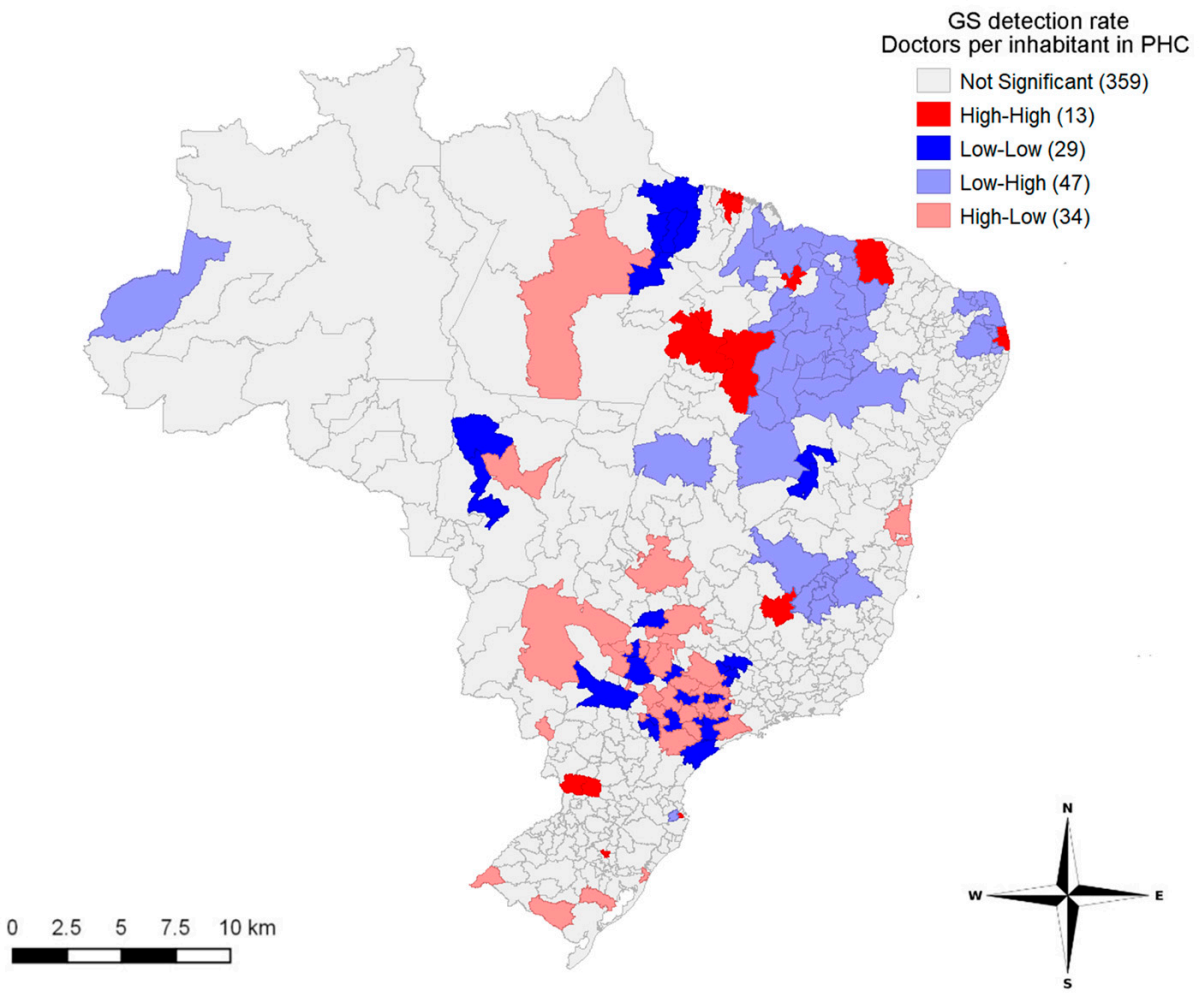
Spatial distribution of gestational syphilis (GS) detection rate and proportion of doctors per inhabitant in primary health care (PHC) in immediate regions of urban articulation. Values in parentheses represent the number of immediate regions of urban articulation with a *P* value ≤ 0.05. Brazil, 2008 to 2018.

The spatial pattern of correlations between the GS detection rate and the proportion of physicians in PHC per inhabitants in the IRUAs (Moran’s I = −0.164, *P* ≤ 0.05) are shown in Figure 5. Corroborating with the clusters displayed in Figure 4, Figure 5 shows clusters with a high rate of detection of GS and a low proportion of physicians in PHC observed in the North, and in the states of São Paulo and Rio Grande do Sul. The cluster formed in Figure 5, covering the Midwest region, also deserves attention, with a high detection rate for gestational syphilis and a low proportion of PHC physicians per population. It is important to emphasize that clusters were formed in the Northeast, in the state of Minas Gerais and on the border between the state of Amazonas and Peru, showing areas with low rates of GS and greater proportions of physicians per inhabitant in PHC.

## DISCUSSION

Our study used spatial analysis to measure the magnitude of GS as a public health problem in several Brazilian regions. Syphilis formed clusters in different regions and was correlated with social variables and PHC coverage, demonstrating areas of high vulnerability.

The Midwest presented more high–high clusters of GS. A study^14^ of syphilis in this region indicated that the population from cities with international borders with Paraguay and Bolivia were exposed to a greater risk of contracting sexually transmitted infections (STIs), which may have contributed to the high transmission rate of syphilis. Illegal drug trafficking in that area may also have favored the vulnerability of the population. Another study^15^ observed an increased GS detection rate in a state in the Midwest with great economic development, corroborating the results of our study. The region studied by the authors had an important industrial food hub and extraction of minerals, which may have contributed to migration and a floating population, and facilitated STI transmission. In this sense, we suggest that the immigration process may have some influence on the syphilis detection rate, as well as on its transmissibility dynamics. Researchers^16^ propose that strategies adopted to reduce the burden of STIs should take into account the relatively high residential mobility of at-risk populations to reduce the spread of infections to new areas.

The presence of major highways in the region may also increase STIs and form high–high clusters of GS. Although the Cuiabá-Santarém highway (BR-163) that crosses the states of Mato Grosso and Mato Grosso do Sul in the Midwest is important for the economy, it facilitates the migratory process, use of drugs, fluctuation of the male population, and sex trade, favoring STI transmission.^17^ In this region, we observed a spatial pattern of high rates of GS and a low proportion of doctors per inhabitant in PHC, indicating that human resources in PHC and strategies for reorganizing the PHC are essential for syphilis control.

Regions of urban articulation in the South also showed high–high clusters of the GS detection rate, and a spatial pattern of high rates of syphilis and MHDIs. This may be explained by better access to health services in populations of more developed regions (e.g., South and Southeast), increasing the number of notifications.^18^ de Oliveira et al.^15^ reinforced that syphilis was not limited to populations with low social conditions because it may also spread in areas with easy access to education. Therefore, actions to control syphilis need population-wide policies for different contexts throughout the country. The high syphilis detection rate and low percentage of PHC coverage also indicate the need to strengthen PHC in the South. Studies^19^^–^^21^ developed in this region highlighted obstacles in prenatal care that may have influenced the increased GS detection rates. Furthermore, difficulties managing the treatment of pregnant women and sexual partnerships were identified, reinforcing the need for improving prenatal care.

Clusters of GS in RUAs of the Southeast were contradictory: high rates of syphilis and high MHDIs, and low rates of syphilis and high MHDIs. The former correlation may have occurred because regions with high MHDIs offer great access to diagnostic methods and present a better notification system.^18^^,^^22^ In the latter situation, regions with high MHDIs, present great service conditions, which may improve access to treatments.^18^ Regions of urban articulation located in the Southeast that formed clusters of high GS detection rates and a low proportion of doctors per inhabitant in PHC may indicate obstacles in the PHC. Furthermore, clusters in RUAs in the Southeast presenting high GS detection rates and a low percentage of PHC coverage reflect a weakness in PHC. Access to PHC may also be difficult and may increase the rate of GS in the Southeast. The state of São Paulo, located in the Southeast region, concentrates the highest proportion of doctors per inhabitant (including all medical specialties), valuing the individual clinic and strengthening secondary and tertiary care in the health care network.^23^

A low–low spatial pattern of the GS detection rate was found in the Northeast. The most plausible explanation for these findings is the greater PHC coverage, because this region has a high social vulnerability. Our results corroborate studies that showed a negative correlation between GS detection rate and Family Health Strategy coverage,^24^ reinforcing the importance of PHC for treatment, health promotion, and disease prevention. The maps with geographic distributions of clusters indicated that this region had a high proportion of doctors per inhabitant in PHC and low GS detection rates. Data also suggest the importance of correlations between the availability of doctors in PHC and actions of detection, treatment, and prevention of syphilis. Primary health care is important because it is the entry and communication center of the health-care network. Moreover, it can be resolute and offer services and universal access in the Brazilian Unified Health System based on integral care and inter- and multidisciplinary work.^25^^,^^26^

Despite the low rates observed in the Northeast, GS is still far from being controlled or reaching the rates recommended by the WHO (i.e., < 0.5 cases/1,000 live births). We also highlight the possibility of underreporting problems in the surveillance systems of the area.^27^ Machado et al.^28^ indicated social vulnerability and difficulties managing resources in the health sector of the Northeast, which may hamper the control of syphilis. Furthermore, social and economic factors in the region were correlated with cases of GS, according to de Macêdo et al.^29^ and de Conceição et al.^30^

Syphilis detection rates were greater in RUAs of the North than in the Northeast; however, no statistical significance was observed compared with national data. These data led us to believe in underreporting records, because this region faces social vulnerability and limited PHC coverage. Freitas et al.^22^ found that women from Brazilian cities with low MHDIs were less likely to have access to tests for diagnosing syphilis. Spatial clusters formed in RUAs of the North by correlating the GS detection rate and MHDIs highlight the relationships between GS and social vulnerability. This index was comprised of dimensions of longevity, education, and income, and was presented coherently in the territory because several regions had high syphilis detection rates and low MHDIs. Our study also showed spatial clusters formed in RUAs of the North, indicating high GS detection rates and a low proportion of doctors per inhabitant in PHC and percentage of PHC coverage. These findings agree with those of Soares Filho et al.,^31^ who indicated the Brazilian North as a critical area according to the proportion of Family Health Strategy teams per inhabitant. Another study^32^ demonstrated the unavailability of medical appointments and lack of doctors in a city in the North. According to de Oliveira et al.,^23^ the poor distribution of doctors in the country varies according to region, medical specialty, and level of care; the PHC faces difficulties in keeping professionals in this health care network.

In this sense, a government plan for encouraging and maintaining doctors in these areas is needed to strengthen PHC and control the syphilis epidemic. The low proportion of PHC units in the North associated with geographic factors in the region increases difficulties related to access to health services and may contribute to a negative scenario of syphilis. Geographic characteristics of other countries (e.g., Australia) also led to difficult access to health services by the population.^33^

It is important to point out that clusters with a high GS detection rate, high PHC coverage, and a high proportion of PHC physicians per population may reflect women’s improved access to health services. Thus, the functionality of the health system can culminate in greater opportunities to offer exams for the diagnosis of syphilis, and more testing, which may contribute to the increase in the number of notifications of the condition in certain areas.^34^

However, it is possible that, in some areas, underreporting and lack of availability of tests for diagnosing syphilis also occur in health services with high PHC coverage and a high proportion of PHC physicians per population, which are reflected in a low GS detection rate and flaws in the quality of prenatal care.^35^

Our study may present limitations because of the use of secondary data from Brazilian information systems and possible underreporting. However, no major changes were observed in the context of the country. The use of data up to 2018 was a result of availability in the SINAN during data collection. Future studies using spatial analysis and focusing on regions of epidemiological interest (e.g., high–high detection rate clusters of GS), and other variables and forms of aggregation are needed.

## CONCLUSION

The spatial analysis of GS in Brazil showed high–high clusters of GS detection rates in the South and Midwest. On the other hand, the central area of the Northeast showed low–low clusters. The correlation between GS detection rate and MHDI indicated the influence of socioeconomic aspects in several areas of the country. Clusters of health-care variables showed a negative correlation between rates of GS in certain areas and PHC coverage, proportion of doctors, and basic health units per inhabitant in PHC.

Specific regions need to reorganize the health-care network, improve PHC coverage, encourage incentive policies, and maintain doctors in PHC. Our study may contribute to establishing and reorienting public policies to control GS in different contexts of the Brazilian territory. Implementing actions, ensuring equity and comprehensiveness in health, and granting access to adequate detection, treatment, and monitoring of GS may help improve its control in Brazil.

## Supplemental Material


Supplemental materials
