# Supplementary material for: Spatial Distribution of Gestational Syphilis in Brazil: Socioeconomic and Health Services Inequalities
Source: Am J Trop Med Hyg. 2023 May 15;109(1):42–9. doi: 10.4269/ajtmh.22-0449 (PMC10324014; doi:10.4269/ajtmh.22-0449)
Supplement: Supplementary file 1 [file tpmd220449.SD1.pdf]

SUPPLEMENTARY MATERIAL

Table S1 - Characteristics of dependent and independent variables for assessing the spatial distribution of gestational syphilis between 2008 and 2018 in Brazilian Regions of Urban Articulation.

	VARIABLES	CONCEPTUALIZATION	DATA SOURCE
DISEASE	Gestational syphilis detection rate	Number of reported cases of gestational syphilis, divided by the total number of live births in a given period and place of residence, and multiplied by 1,000.	DATASUS (SINAN, SINASC)<br><br>Number of reported cases of gestational syphilis<br>DATASUS Health information (Tabnet) Epidemiological and Morbidity Notifiable Diseases and Conditions – from 2007 onwards (SINAN) Gestational syphilis Brazil by city City of residence Year of diagnosis Period from 2008 to 2018<br>http://tabnet.datasus.gov.br/cgi/tabcgi.exe?sinannet/cnv/sifilisgestantebr.def<br>Accessed on June 25, 2021<br><br>Total number of live births<br>DATASUS Tabnet Vital Statistics Live births – from 1994 to 2019 Live births Birth by residence of the mother<br>http://tabnet.datasus.gov.br/cgi/tabcgi.exe?sinasc/cnv/nvbr.def<br>Accessed on June 25, 2021
ECONOMIC AND SOCIAL	MHDI	Geometric mean of income, educational level, and longevity dimensions (equal weights).	PNUD<br>AtllasBrasil Collection Library Data Source Demographic census 2010<br>https://onedrive.live.com/?authkey=%21ABiV0mb1HeyuOxU&cid=124653557C0404EC&id=124653557C0404EC%2123017&parId=124653557C0404EC%2122899&action=locate<br>Accessed on June 29, 2021
HUMAN RESOURCES	Proportion of doctors per inhabitant in the primary health care	Number of specific professionals in a given location and period, divided by the population of that location and period, and multiplied by 3,500<br><br>3,500 is the maximum number of people covered by the professional according to the Ordinance 2,436/2017 of primary health care	CNES<br>DATASUS Tabnet Assistance network CNES - Human resources from august 2007 - Occupations classified by the CBO 2002 Professionals Medical Doctors of Family Health Strategy, Family and Community Doctor Answer on SUS Dez/2008, Dez/2009, Dez/2010, Dez/2011, Dez/2012, Dez/2013, Dez/2014, Dez/2015, Dez/2016, Dez/2017, Dez/2018<br>http://tabnet.datasus.gov.br/cgi/tabcgi.exe?cnes/cnv/prid02br.def<br>Accessed on June 28, 2021

ACCESS	Percentage of primary health care coverage teams	Estimated population covered in the primary health care (i.e., percentage of the population covered by Family Health Strategy teams and equivalent traditional primary health care teams, normalized to the population estimative).	Primary Care e-Manager<br>Primary Health Care Coverage Search options Geographic Unit by Period □ Cities All regions, All states, and All cities Competence December/2018.<br>https://egestorab.saude.gov.br/paginas/acessoPublico/relatorios/relHistoricoCoberturaAB.xhtml<br>Accessed on June 26, 2021.

MHDI: Municipal Human Development Index; DATASUS: Department of Informatics of the Unified Health System; SINAN: Notifiable Diseases Information System; PNUD: United Nations Development Program; SINASC: Live Birth Information System; CNES: National Registry of Health Establishments; CBO: Brazilian Classification Occupation Classification.
